# Comparison of Aerosol Pt, Au and Ag Nanoparticles Agglomerates Laser Sintering

**DOI:** 10.3390/ma15010227

**Published:** 2021-12-29

**Authors:** Kirill Khabarov, Messan Nouraldeen, Sergei Tikhonov, Anna Lizunova, Olesya Seraya, Emiliia Filalova, Victor Ivanov

**Affiliations:** Moscow Institute of Physics and Technology, National Research University, 141701 Dolgoprudny, Russia; messannouraldeen@phystech.edu (M.N.); sergei.s.tikhonov@phystech.edu (S.T.); Lizunova.aa@mipt.ru (A.L.); seraia.ov@phystech.edu (O.S.); filalova.em@phystech.edu (E.F.)

**Keywords:** aerosol nanoparticles, platinum, gold, silver, spark discharge, pulsed laser radiation, IR, shrinkage, plasmon resonance, extinction

## Abstract

In this paper, we investigated the interaction of nanosecond pulsed-periodic infrared (IR) laser radiation at a 50 and 500 Hz repetition rate with aerosol platinum (Pt) and silver (Ag) nanoparticles agglomerates obtained in a spark discharge. Results showed the complete transformation of Pt dendrite-like agglomerates with sizes of 300 nm into individual spherical nanoparticles directly in a gas flow under 1053 nm laser pulses with energy density 3.5 mJ/cm^2^. Notably, the critical energy density required for this process depended on the size distribution and extinction of agglomerates nanoparticles. Based on the extinction cross-section spectra results, Ag nanoparticles exhibit a weaker extinction in the IR region in contrast to Pt, so they were not completely modified even under the pulses with energy density up to 12.7 mJ/cm^2^. The obtained results for Ag and Pt laser sintering were compared with corresponding modification of gold (Au) nanoparticles studied in our previous work. Here we considered the sintering mechanisms for Ag, Pt and Au nanoparticles agglomerates in the aerosol phase and proposed the model of their laser sintering based on one-stage for Pt agglomerates and two-stage shrinkage processes for Au and Ag agglomerates.

## 1. Introduction

The optical and electrical properties of metal nanoparticles (NPs) are interrelated and largely determined by the electronic configuration of the material and their sizes [1]. For example, in NPs consisting of hundreds–thousands of atoms, electronic states become discrete due to the electron mean free path (MFP) limiting at the surface and grain boundaries [2,3]. For this reason, the spectral characteristics and some parameters as electrical conductivity and magnetic susceptibility of such NPs change significantly and exhibit quantum-dimensional effects [4]. Significant nonlinearity in the parameters of nanoscale metals is also observed for larger dimensions of electrons’ spatial localization [5]. Simultaneously with the nanoobjects’ size parameters, the mobility of electrons is determined by the material electronic configuration affecting the intrinsic MFP of charge carriers. For example, the electrons MFP for platinum group metals (Pt, Ir, Pd, Ru) turn out to be shorter than for noble metals (Au, Ag, Cu). As a result, such metals may show lower resistivity in nanoscale due to less scattering at the nanoobjects surface and grain boundaries [5]. In this regard, the study of the spectral characteristics of isolated NPs, severely limited in size in any of the directions, is an urgent task [6,7].

Recently, NPs properties have been studied by spectrophotometric approaches, e.g., X-ray photoelectron spectroscopy (XPS) [8], ultraviolet photoelectron spectroscopy (UPS) [9] and others. However, the results of the presented methods’ research significantly depends on the environment inevitably interacting with NPs, and this plays a crucial role. For instance, the electronic structure of NPs changes greatly in colloidal solutions, which leads to significant shifts in optical resonances and difficulties in obtaining accurate results [10]. This creates the need to develop approaches for the study of NPs in a gas phase. These approaches are promising for obtaining high-precision results that are in good agreement with the theory. In our previous work [11], we have already proposed such an approach and studied optical spectra and processes of laser radiation effect on Au NPs agglomerates expressed in their sintering directly in a gas flow. However, the comparison of this metal with Pt and Ag is also of great interest and defines the novelty of the work considering the sintering mechanisms of nanoparticles right in the aerosol phase. These materials are of fundamental importance for a variety of catalytic [12] and plasmonic [13] applications such as photocatalysis [14,15,16], synthesis of reactive oxygen species [17,18], imaging [13,19], detection of chemical and biological substances [20], and others.

In this paper, we experimentally studied the extinction cross-section spectra and sintering processes by pulsed nanosecond laser radiation with a wavelength of 1053 nm for aerosol agglomerates of NPs synthesized in a spark discharge (SD). Agglomerate sintering is caused by the diffusion flow of matter in a direction that provides minimal surface energy, which can be achieved with a spherical shape [21]. To do this, we used a previously developed laser modification cell [11], which combined the studied aerosol flow with the optical axis of impacting radiation. In the experiments, the NPs agglomerates were spatially separated—their interactions with each other and with the experimental setup were minimized. The study was done for Pt and Ag NPs materials significantly differ in optical and electrical properties. Here we measured the NPs agglomerates’ average size and concentration online by their differential electrical mobility during their laser modification, depending on the energy density of laser radiation pulses. Along with laser exposure, we studied the extinction cross-section of these NPs agglomerates in a wide spectral range by a spectrophotometer. The obtained results for Pt and Ag were compared to results newly obtained for Au NPs (TEM images, NPs distribution and extinction cross-section spectra) and taken from our previous work (size-energy density dependencies) [11].

## 2. Materials and Methods

Primary NPs were synthesized in the SD during the electrical erosion of the electrodes in an atmosphere of high purity argon (99.9999%) at an excess gas pressure of 0.6 atm. The operation principles of a NPs SD generator with all the explanations and parameters of the electrical scheme used are described in detail in [22]. The electrodes used in the work were made of Pt, Au and Ag with a purity of 99.9999% and had a shape of hollow cylinders with an outer diameter of 8 mm (Plaurum Group, Verhnyaya Pyshma, Russia). In the case of Pt and Au, the thickness of the cylinder wall was 1 mm, and, in the case of Ag, 3 mm. The high purity of the metal and gas medium together with the vacuum tightness of the gas path and synthesis chamber ensured the maximum possible purity of the studied NPs during the experiments.

The study of the optical radiation interaction with aerosol NPs agglomerates was carried out in the cell discussed in detail in [11]. In the experiments, we used a pulsed laser with the wavelength of 1053 nm (TECH-1053, “Laser-export” Co. Ltd., Moscow, Russia) with a pulse duration of about 40 ns and controlled pulse repetition rates in the range of 10 Hz–10 kHz. Here we conducted the experiments at the pulse repetition rates of 50 and 500 Hz with equal pulse energy range up to 900 µJ.

**Figure 1:**
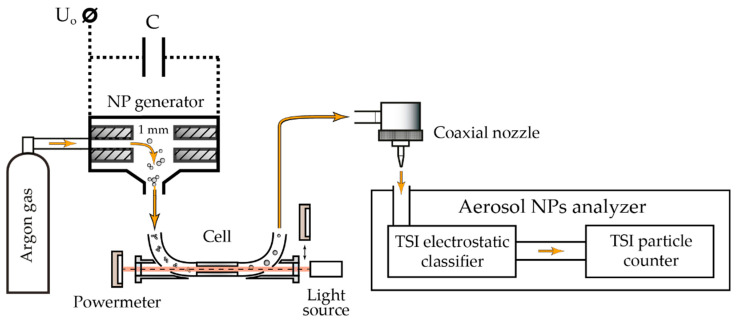
The scheme of the experimental setup.

Schematically, the experimental setup is shown in Figure 1.

In all the experiments, the laser was initially set up at the position of the light source in front of the cell so that the radiation was directed opposite to the aerosol flow. The gas flow rate used (Q = 50 mL/min) determined the velocity of NPs inside the cell υ = 117.9 mm/s. The diameter of the laser beam in the cell was 3 mm. The study of the laser radiation interaction with NPs was done under the control of the average radiation power by the thermal power meter PD300-3W-V1 (Ophir, North Logan, UT, USA). The energy density of a single laser pulse was calculated based on the measured average radiation power and known pulse repetition rate, provided that the laser generates the same pulses [11].

The optical spectra of aerosol NPs agglomerates were studied in the wavelength range of 300–1000 nm by the spectrophotometer HR4000CG-UV-NIR (Ocean Insight, Orlando, FL, USA) with a white light source (halogen lamp), equipped with an optical fiber, that was installed in front of the cell. For this purpose, for each material, we did successive measurements of the halogen lamp spectrum through the cell, initially filled only with the gas, and then with the aerosol. The measurements were done with the fixed geometry of the experiment and stable position of the optical elements. Final spectra of aerosol NPs were obtained by normalizing spectra for the cell filled with the aerosol on spectra for the cell filled with the gas only. This procedure excluded the influence of possible reflections of radiation inside the cell on the results.

**Figure 2:**
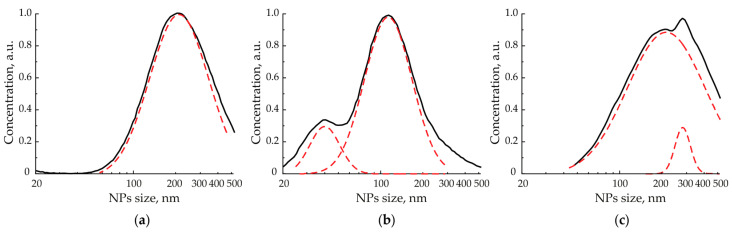
Examples of size distributions for initial (**a**) and laser modified (**b**,**c**) NPs agglomerates (black curves) with their log-normal multiple peak approximation (dashed red curves). Here, the NPs size axis is provided in the logarithmic scale.

Statistical size distributions of aerosol NPs, as well as their total concentrations in the gas flow, were controlled online after the particles left the cell through a focusing nozzle by the direct measurements of the aerosol NPs analyzer SMPS 3936 (TSI Inc., Shoreview, MN, USA) based on their differential electrical mobility. The statistical size distributions of aerosol NPs obtained in these measurements were described by a log-normal distribution. To construct the dependences of the agglomerates size on the energy density of laser pulses, we approximated the size distributions with a set of log-normal peaks, controlling R^2^ > 0.95, and measured the maxima of the highest ones (Figure 2). The focusing nozzle with an outlet diameter of 300 µm simultaneously delivered NPs to the analyzer and stabilized the parameters of pressure and flow of the carrier gas. It is worth mentioning that possible losses of NPs, due to their deposition on walls of a pipe between the cell and the analyzer with a length of about 200 mm, caused by the diffusion processes, weakly affecting the concentration measurements, are less than 3% and are within the error of the analyzer [11].

Additional studies of shapes and sizes of NPs were done based on images obtained by the transmission electron microscope (TEM) JEM-2100 (JEOL, Ltd., Tokyo, Japan). To do this, NPs were collected on TEM grids installed at the focusing nozzle outlet right after the cell. According to the set of obtained TEM images, we plotted size distributions and measured modal sizes for primary synthesized NPs in the agglomerate composition and for spherical NPs sintered by laser radiation for the three metals by processing 600 pieces on average, approximating them with spheres.

## 3. Results

### 3.1. NPs Spectral Characteristics

**Figure 3:**
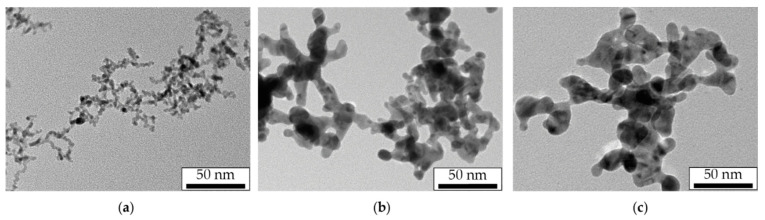
TEM images of Pt (**a**), Au (**b**) and Ag (**c**) NPs agglomerates.

During the transportation in the aerosol flow, primary NPs synthesized in the SD experience many collisions due to their Brownian motion and assemble into dendrite-like agglomerates of arbitrary shape characterized by a log-normal size distribution with a modal size of 150–300 nm (Figure 3). The agglomerates size depends on the velocity parameters of the aerosol determining the NPs flow time, initial NPs concentration, and the sizes of primary NPs for each material. In our study, the modal sizes of Pt, Au and Ag agglomerates measured by the aerosol spectrometer were 300, 280 and 167 nm, respectively. At the same time, the modal sizes of primary NPs were analyzed from a series of TEM images and were found equal to 3.5, 7.5 and 17.5 nm for Pt, Au and Ag, respectively. As a result, Au and Ag NPs agglomerates had larger necks connecting primary NPs.

**Figure 4:**
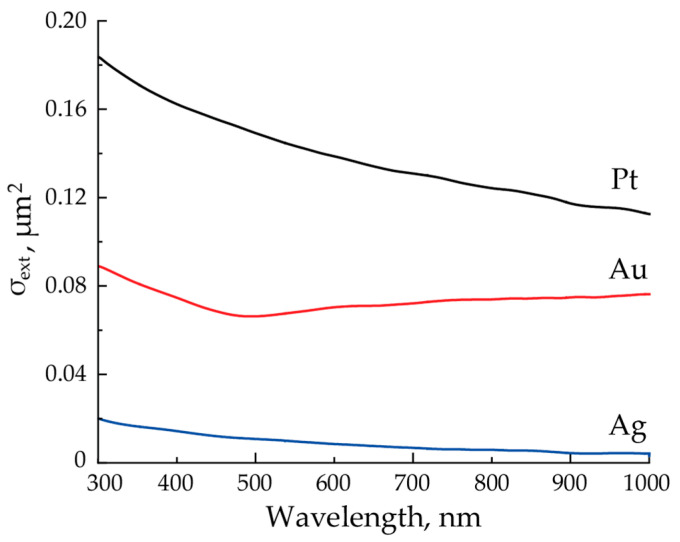
Extinction cross-section spectra of a single NP averaged over the flow of initial agglomerates for Pt, Au and Ag.

Aerosols of initial agglomerates were studied by the spectrophotometer for all the three materials. Based on the obtained transmission spectra for the initial agglomerates, we determined the extinction cross-section spectra of one agglomerate (Figure 4), averaged over the aerosol flow, calculated according to the Beer–Lambert law [23].

We also determined the extinction cross-section of initial agglomerates at the wavelength of 1053 nm radiating them by laser pulses with the energy density 3 mJ/cm^2^ and pulse repetition rate of 500 Hz at the gas flow rate 200 mL/min. With these parameters, the agglomerates were still unmodified. Extinction cross-sections equaled 0.105, 0.072 and 0.005 µm^2^ for Pt, Au and Ag, respectively, which is comparable to the literature data for nanostars studied in colloidal solutions [24]. Neglecting the fact that the agglomerates size could slightly change due to the change in the gas flow, in the further discussion, we will use these cross-sections. With comparable sizes of NPs agglomerates for Pt and Au, Pt agglomerates had the largest extinction cross-section, and the extinction cross-section of Ag NPs turned out to be the smallest. The result does not change even if we take into account the less than 2 times smaller modal size of Ag NPs agglomerates, since their extinction cross-section is 21 and 14 times smaller than for Pt and Au at the laser wavelength, respectively.

### 3.2. NPs Agglomerates Laser Modification

We studied the effect of nanosecond pulse-periodic laser radiation with the wavelength of 1053 nm on the size of sintered NPs agglomerates depending on the pulse energy density at the repetition rates of 50 and 500 Hz. The sizes were measured in the gas flow by the NPs electrical mobility. Similarly to [11], we will call the sintering of NPs in the aerosol flow complete if, as a result of their modification, only spherical particles remain, which does not change with a further increase in the pulse energy density. Otherwise, we will consider the sintering process incomplete.

**Figure 5:**
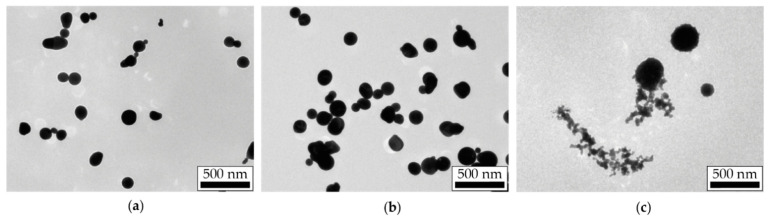
TEM images of Pt (**a**), Au (**b**) and Ag (**c**) NPs agglomerates, modified by laser radiation with the wavelength of 1053 nm at the maximum pulse energy density and pulse repetition rate of 500 Hz for the aerosol flow rate 50 mL/min.

The morphology of NPs modified by laser radiation at the maximum pulse energy density and pulse repetition rate of 500 Hz is shown in the TEM images presented in Figure 5. Under the close sintering conditions, the Pt and Au agglomerates were completely sintered, which is confirmed by the large spherical NPs in Figure 5a,b. However, for Ag, one can see dendritic structures in the TEM images (Figure 5c), which characterize the sintering as incomplete.

**Figure 6:**
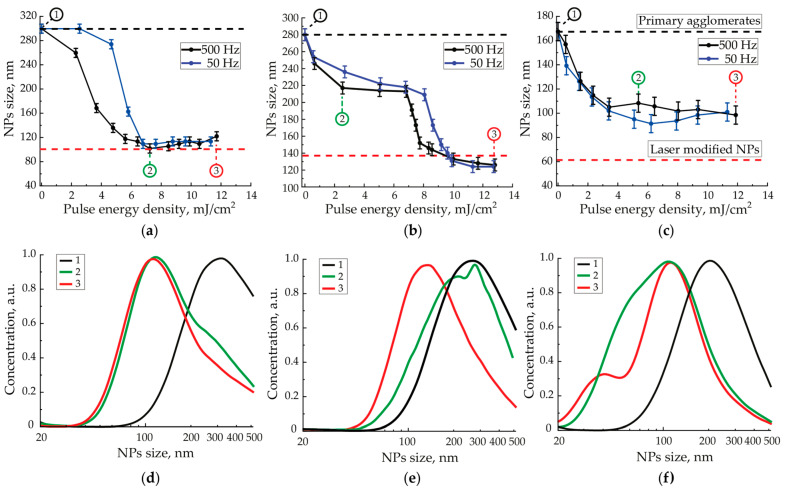
Dependencies of Pt (**a**), Au (**b**) and Ag (**c**) NPs size at the output of the focusing nozzle right after the cell on the pulse energy density of laser radiation with the wavelength of 1053 nm at the pulse repetition rate of 500 Hz and the aerosol flow rate of 50 mL/min. The shrinkage curve data for Au is from [11]. Size distributions of initial and laser modified Pt (**d**), Au (**e**) and Ag (**f**) NPs agglomerates taken from 1, 2 and 3 measurements presented in (**a**–**c**).

During the pulsed laser sintering of aerosol NPs agglomerates, we measured the sizes of sintered NPs in the stream by their electrical mobility, depending on the energy density of the incident radiation pulse (Figure 6a–c). In this figure, the black dashed line corresponds to the distribution modal size of the initial agglomerates, determined by their electrical mobility. The red dashed line corresponds to the modal size of sintered agglomerates determined by statistical processing of the part of sintered spherical NPs on a series of TEM images for each of the three materials. As for Ag NPs, the measured modal size may slightly differ from the actual modal size of completely modified NPs and is shown on the graph for result comparison. This is shown by the small red peak in Figure 6f with a maximum of around 40 nm.

Experimental dependencies of the NPs agglomerates’ size for the three metals at the exit from the cell (Figure 6) demonstrate a decrease in the size as the energy density of laser pulses increases. This process should be called NPs shrinkage as the result of laser sintering in analogy with the shrinkage of macroscopic powder green bodies under the energy exposure to them [25].

On the presented curves corresponding to Pt and Au, one may observe an S-shaped behavior with an initially slow decrease in size to a certain threshold value of the pulse energy density, then with a rapid decrease in size in a narrow energy density range and, finally, a slow decrease with an output to a constant value. However, on similar dependencies for Ag NPs, the rapid size reduction in a narrow energy density range was not achieved, and sintering turned out to be incomplete. This is also confirmed by the TEM images of laser modified Ag NPs (Figure 5c).

## 4. Discussion

The shrinkage of Pt NPs agglomerates caused by laser radiation, shown in Figure 6a, is characterized by a slow decrease in size at the pulse energy density values below 2.5 and 4.5 mJ/cm^2^ at the pulse repetition rates of 500 and 50 Hz, respectively. With a further increase in the pulse energy density, a steep shrinkage interval is observed, reaching a horizontal section with a final NPs size of about 100 nm at the pulse energy densities of 6.0 and 6.7 mJ/cm^2^ and pulse repetition rates of 500 and 50 Hz, respectively. According to aerosol in-flow particle size analysis, the unimodal agglomerate distribution was observed for three main stages of the Pt shrinkage process: 1st at the beginning of the experiments when measuring initial agglomerates, 2nd after the end of the first step of sintering, when the shrinkage curve flattened for the first time, 3rd at the maximum pulse energy density observed in our experiments. Comparing Pt NPs size distributions, we discover a 200 nm-shift of the unimodal peak to the low-size range with the growth of the energy density (Figure 6d). One may find in thermal sintering of powder green bodies prepared from identical particles with a unimodal size distribution a similar one-stage shrinkage process with one section of steep size change. In this case, particle sintering requires close energies [25]. Based on the presented analogy and the presence of only one type of agglomerates, we can assume that the absorption characteristics of radiation at the wavelength of 1053 nm are close for all Pt NPs agglomerates.

It is worth noticing that Pt NPs agglomerates require the lowest pulse energy density for complete laser sintering among other materials. This can be explained by a comparison of optical properties for metal NPs. Small Pt NPs have an approximately constant high-level absorption 0.4 in red and IR range starting from around 500 nm wavelength [26]. Close results were received in a simulation of optical radiation absorption by thin Pt films with a thickness of about 10 nm on glass surfaces [27]. In the above-noted work, the thin films were characterized by a high and stable absorption of about 0.4 over a wavelength range of 250–900 nm. Hereafter, because of the lack of experimental and theoretical research on the absorption of IR radiation by NPs, and taking into account the noted data about Pt films and particles, we present the literature data about electrical and optical properties of thin films and consider them close to NPs ones.

Experimental and theoretical studies of the electrical resistivity of polycrystalline Pt films [5] showed that, with a decrease in the thickness of Pt films from 40 to 3 nm, their electrical resistivity increased by less than 2 times. Moreover, Pt thin films showed the lowest resistivity for films with a thickness below 5 nm in comparison with other etalon conductive materials such as copper. This effect was explained by a weaker thickness dependence of both surface and grain boundary electron scattering in Pt and connected with the efficiency of absorption.

In contrast to Pt, the interaction process of IR laser radiation with Au agglomerates was characterized by a clearly expressed two-stage shrinkage with two areas of steep size change (Figure 6b) from our work [11]). The first section of steep shrinkage starts at the minimum values of the energy density of laser pulses and ends at about 2.5 and 5.0 mJ/cm^2^ at the pulse repetition rates of 500 and 50 Hz, respectively. The second steep shrinkage phase is observed in the ranges of pulse energy densities 7.0–8.0 mJ/cm^2^ and 8.3–10.0 mJ/cm^2^ for the pulse repetition rates 500 and 50 Hz, respectively. The shift of a steep section of the shrinkage curves towards higher values of the energy density for both Au and Pt caused by the reduction of the pulse repetition rate is associated with a discrete-stepwise process of NPs shrinkage under the influence of laser pulses. In particular, this happens because of the fewer number of interacting pulses requiring a higher energy density to modify NPs [11]. Two-stage shrinkage processes were also observed during thermal sintering of powder green bodies prepared from particles with a two-modal size distribution [28], as the particles with a smaller characteristic size require less energy for sintering than large particles.

During the analysis of the evolution of Au and Ag NPs size distribution, we found that initially unimodal agglomerates distribution at the end of the first sintering step splits into a bimodal one (Figure 6e,f). This phenomenon is relative to the formation of two agglomerates fractions with different sizes, and we suggested that some of the initial agglomerates have been completely sintered at this step. The difference in the absorption efficiency of IR radiation between large agglomerates and metal nanospheres explains the observed effect. In [29], the authors have shown that, during the agglomeration process, the plasmon peak around 526 nm of separate Au NPs decreased rapidly. Simultaneously, agglomerates exhibited several peaks, red-shifting, merging and overlapping while the agglomerate size grew. This resulted in the raising and flattening of the absorption in the visible and IR spectrum range. In addition, the growth of the number of NPs in agglomerates composition from 2 to 15 led to a two times increase of IR absorbance, whereas changing the size of agglomerate from 15 to 300 particles resulted in more than three times enlargement of IR absorbance. In addition [30], the absorbance of branched Ag nanostars was characterized by a higher IR absorption comparably to nanospheres with the diameter of 20 nm and differed for stars with various branches lengths [31]. Thus, we suggest that our aerosol ensemble of initial Au and Ag NPs agglomerates consists of two types of agglomerates with different IR absorbance efficiency at the wavelength of 1053 nm that is associated with their different fractal structure.

This assumption is also confirmed by previous studies of optical properties of thin polycrystalline films. In particular, in [32], the optical losses of radiation with wavelengths of 250–2000 nm increased significantly with the reduction of the film thickness from 80 to 20 nm and of crystallites size in their composition. The two-stage shrinkage of Au NPs agglomerates observed in our results may be caused by a steep dependence of optical absorption on the agglomerate shape and size of NPs in the agglomerate that can lead to a more efficient laser heating of smaller or branchy particles. Moreover, Au NPs agglomerates were sintered completely at higher radiation energy densities in comparison with Pt due to a smaller level of optical radiation absorption in the near-IR spectrum region. Earlier, in [27], the authors registered the maximum relative optical absorption for Au films with a thickness of about 10 nm at the level of 0.07 in this spectral range. This value is close to a 0.03 absorption level, which was measured for 20–30 nm Au nanospheres in an IR spectrum [33].

Although Ag also belongs to the group of noble metals as well as Au, and the properties of their bulk materials are similar, in our experiments, we found a significant difference in the shrinkage behavior of Ag NPs agglomerates under the influence of pulsed IR laser radiation. Only one section of steep shrinkage was observed in the dependencies (Figure 6c), which started at the minimum pulse energy density values and ended at about 4.0 mJ/cm^2^ for both 500 and 50 Hz laser pulse repetition rates. Moreover, a deep size shrinkage of about 1.7 times in this energy density range still was still far from the red dashed line corresponding to the quasi-completely sintered NPs. The incomplete sintering was additionally confirmed by the TEM images of NPs modified with the maximum pulse energy density (Figure 5c). With the further increase in the energy density to 12.0 mJ/cm^2^, agglomerate shrinkage practically did not occur.

Analyzing Au and Ag agglomerates distributions in different sintering stages, one initial peak divides into two peaks with close maximums, which are presumably connected to different types of agglomerates (Figure 6e,f). Moreover, the magnitudes of the peaks continuously changed. While the energy density increased, the concentration of completely sintered agglomerates with lower sizes increased and consequently the number of large agglomerates, which have not been sintered yet, decreased. This led to the segregation and growth of a peak for already sintered agglomerates. After laser interaction with Pt and Au agglomerates, we observed the complete sintering of materials that accompanied lognormal size distribution function conservation. However, in intermediate stages, after the sintering of first type of agglomerates, the lognormal unimodal distribution could be transformed to a multimodal one due to the non-equilibrium process of the energy transfer and the difference in IR laser radiation interaction with agglomerates of various shape and crystallites of different sizes. Thus, within the framework of a single-stage shrinkage of Pt, the sintering was done with the conservation of a lognormal distribution, and within the framework of a two-stage shrinkage of Au and Ag, in an intermediate stage, we observed a change in the distribution function and incompleteness of sintering.

Taking into account the bimodal size distribution of Ag NPs at the energy density of 12 mJ/cm^2^ compared to Au, we may conclude that, by applying a higher energy density of laser pulses with the wavelength of 1053 nm, the complete sintering of agglomerates can be achieved. Unfortunately, it could not be realized with our laser, so the second section of steep shrinkage of agglomerates, leading to complete sintering of all Ag NPs, is expected. A large difference in the pulse energy density (more than 8.0 mJ/cm^2^) to the expected second shrinkage step may say that Ag nanocrystals and agglomerates perform a strong dependence of optical radiation absorption on their size with a small absolute absorption value. Indeed, similar data were previously obtained in several papers. Thus, in [34], it was shown that the Ag film with the thickness below 8 nm displayed a non-metallic behavior in the wavelength range from 600 to 1000 nm. When the thickness was larger than 12 nm, the extinction coefficient was stable and became closer to those of bulk Ag. Moreover, when the film thickness changed from 6.0 to 4.7 nm, the extinction coefficient decreased by more than 10 times. In addition, in [27], for Ag films with a thickness of about 10 nm optimized for maximum absorption, a relative optical absorption in the near-IR range below 0.01 was found, which was about seven times less than the absorption of Au under similar conditions.

The sintering results of aerosol Pt, Au and Ag NPs agglomerates by nanosecond laser pulses of 1053 nm together with the results of other authors are in good agreement with that determined in our experiments’ extinction cross sections for such agglomerates, presented in Figure 4. The extinction values for Pt, Au and Ag NPs agglomerates correlate well with the laser radiation complete sintering efficiency. Moreover, at the laser wavelength, the extinction of Au agglomerates is 1.5 times less in comparison with Pt, and the extinction of Ag agglomerates is 14 times less in comparison with Au.

## 5. Conclusions

In this paper, we have studied the laser sintering mechanisms of Pt and Ag NPs agglomerates obtained in the SD. The sintering was done by the nanosecond pulsed-periodic laser radiation with the wavelength of 1053 nm, pulse energy density range up to 12.7 mJ/cm^2^ and repetition rates of 50 and 500 Hz directly in the gas flow. We obtained the shrinkage curves for NPs agglomerates of the two metals (Pt and Ag) that represent the dependence of the modified NPs agglomerates size on the laser pulse energy density and compared them with the one for Au NPs, discussed in our previous work.

In our experiments, we registered the complete modification of NPs agglomerates into separate spherical NPs only for Pt and Au at energy densities above 3.5 and 7.0 mJ/cm^2^ with shrinkage in size by 2.9 and 2.3 times, respectively. Moreover, the shrinkage for Au NPs demonstrated a two-stage laser modification process, whereas, for Pt NPs, this process passed in one stage. Thus, within the framework of a single-stage Pt shrinkage, sintering proceeded with the conservation of a unimodal lognormal agglomerate size distribution with a mode shift only. In contrast, according to a proposed two-stage shrinkage model for Au and Ag, in an intermediate stage, we observed a transformation of the distribution into a bimodal one and incompleteness of sintering for Ag even under the pulses with energy density up to 12.7 mJ/cm^2^. We associated the two-stage process for Au and Ag with the existence of two types of agglomerates characterized by the different IR absorption efficiency connected with the different fractal structure of agglomerates and crystallite size within the agglomerates.

It was found that the energy required for complete laser modification correlates well with the extinction of NPs increasing for materials in the Pt, Au, Ag order. Thus, according to the experimental results, Ag NPs with an extinction coefficient 14 times less than one for Au NPs at 1053 nm have not been completely modified. At the same time, we detected only partial transformation of agglomerates into spheres with the relative shrinkage to the size of 100 nm approximately equal to 1.7 at the energy density of 12.0 mJ/cm^2^.
